# Supplementary material for: Effect of Borrelia burgdorferi on the Expression of miRNAs in Breast Cancer and Normal Mammary Epithelial Cells
Source: Microorganisms. 2023 Jun 1;11(6):1475. doi: 10.3390/microorganisms11061475 (PMC10300985; doi:10.3390/microorganisms11061475)
Supplement: Supplementary file 1 [file microorganisms-11-01475-s001.zip › microorganisms-2422007-supplementary.pdf]

miRname (human)	Order (by row)	Well	Cq 48hr Uninfected (Control)	Cq 48hr Infected (Experimental)
hsa-let-7a-5p	1	A01	20.08	18.29
hsa-let-7b-5p	2	A02	21.36	20.36
hsa-let-7c-5p	3	A03	21.56	19.47
hsa-let-7d-5p	4	A04	22.93	21.22
UniSp2	5	A05	36.22	38.06
hsa-miR-200a-3p	6	A06	31.15	30.44
hsa-let-7g-5p	7	A07	21.87	20.05
hsa-let-7i-5p	8	A08	20.90	19.43
hsa-miR-1	9	A09	37.05	35.16
hsa-miR-100-5p	10	A10	20.34	18.77
hsa-let-7f-5p	11	A11	22.27	20.26
hsa-miR-101-3p	12	A12	24.62	22.29
hsa-miR-103a-3p	13	B01	21.05	19.27
hsa-miR-106a-5p	14	B02	21.65	20.14
hsa-miR-106b-5p	15	B03	22.52	21.09
hsa-miR-107	16	B04	21.29	19.81
UniSp4	17	B05	36.24	32.00
UniSp6	18	B06		
hsa-miR-125b-5p	19	B07	19.62	18.16
hsa-miR-126-3p	20	B08	26.24	24.25
hsa-miR-130a-3p	21	B09	22.61	20.69
hsa-miR-132-3p	22	B10	25.77	24.42
hsa-miR-10b-5p	23	B11	27.57	25.67
hsa-miR-133a-3p	24	B12	34.17	33.11
hsa-miR-141-3p	25	C01	28.19	26.82
hsa-miR-143-3p	26	C02	30.45	30.02
hsa-miR-145-5p	27	C03	29.94	29.35
hsa-miR-146a-5p	28	C04	23.30	20.81
UniSp5	29	C05	37.27	37.55
hsa-miR-26a-5p	30	C06	22.09	20.26
hsa-miR-150-5p	31	C07	30.58	28.46
hsa-miR-155-5p	32	C08	38.35	37.38
hsa-miR-15a-5p	33	C09	20.86	19.21
hsa-miR-15b-5p	34	C10	22.63	20.53
hsa-miR-149-3p	35	C11	30.89	29.39
hsa-miR-16-5p	36	C12	21.08	18.71
hsa-miR-17-5p	37	D01	22.51	20.38

hsa-miR-181a-5p	38	D02	22.02	21.42
hsa-miR-181b-5p	39	D03	23.49	22.14
hsa-miR-182-5p	40	D04	25.20	23.70
cel-miR-39-3p	41	D05		
hsa-miR-27a-3p	42	D06	20.32	18.30
hsa-miR-186-5p	43	D07	26.64	24.49
hsa-miR-18a-5p	44	D08	24.01	22.23
hsa-miR-191-5p	45	D09	22.32	20.64
hsa-miR-192-5p	46	D10	26.13	24.52
hsa-miR-148a-3p	47	D11	28.35	27.12
hsa-miR-194-5p	48	D12	25.81	24.36
hsa-miR-195-5p	49	E01	27.34	25.83
hsa-miR-196a-5p	50	E02	28.37	27.20
hsa-miR-19a-3p	51	E03	20.49	18.68
hsa-miR-19b-3p	52	E04	20.79	19.14
UniSp3 IPC	53	E05	21.78	21.49
hsa-miR-200b-3p	54	E06	30.06	28.51
hsa-miR-200c-3p	55	E07	27.67	26.26
hsa-miR-202-3p	56	E08	36.97	33.30
hsa-miR-10a-5p	57	E09	25.33	24.21
hsa-miR-205-5p	58	E10	37.36	35.53
hsa-miR-206	59	E11	40.41	33.73
hsa-miR-20a-5p	60	E12	21.03	19.53
hsa-miR-20b-5p	61	F01	37.11	34.34
hsa-miR-21-5p	62	F02	16.03	14.49
hsa-miR-210-3p	63	F03	23.16	21.87
hsa-miR-214-3p	64	F04	29.76	26.65
hsa-miR-215-5p	65	F05	26.28	24.43
SNORD38B	66	F06	19.73	18.04
hsa-miR-22-3p	67	F07	20.01	18.95
hsa-miR-221-3p	68	F08	20.62	19.01
hsa-miR-222-3p	69	F09	20.32	18.36
hsa-miR-223-3p	70	F10	33.60	31.57
U6 snRNA	71	F11	18.17	16.01
hsa-miR-23a-3p	72	F12	20.16	18.19
hsa-miR-23b-3p	73	G01	20.05	18.33
hsa-miR-24-3p	74	G02	19.67	18.11
hsa-miR-25-3p	75	G03	22.41	21.09
UniSp3 IPC	76	G04	21.86	21.49
hsa-miR-26b-5p	77	G05	23.69	22.12
UniSp3 IPC	78	G06	21.85	21.72
hsa-miR-27b-3p	79	G07	20.08	18.32
hsa-miR-29a-3p	80	G08	19.97	18.61

hsa-miR-29b-3p	81	G09	20.02	18.97
hsa-miR-29c-3p	82	G10	19.65	17.97
hsa-miR-30b-5p	83	G11	22.57	21.10
hsa-miR-30c-5p	84	G12	21.65	20.21
hsa-miR-30d-5p	85	H01	22.37	20.48
hsa-miR-31-5p	86	H02	28.28	26.98
hsa-miR-34a-5p	87	H03	24.77	23.58
SNORD49A	88	H04	19.59	17.68
hsa-miR-423-5p	89	H05	24.42	23.35
hsa-miR-7-5p	90	H06	24.88	23.39
hsa-miR-9-5p	91	H07	27.39	25.73
hsa-let-7e-5p	92	H08	21.02	19.18
hsa-miR-92b-3p	93	H09	25.98	24.19
hsa-miR-93-5p	94	H10	22.31	20.92
hsa-miR-99a-5p	95	H11	25.50	23.89
Blank (H2O)	96	H12		

MDA-MB-231 - B31-Infected / Uninfected -

Uninfected Cq Reference (SNORD38B)	Infected Cq Reference (SNORD38B)	dCt 48hr Uninfected (Uninfected Cq - Reference Cq)
19.73	18.04	0.35
19.73	18.04	1.63
19.73	18.04	1.82
19.73	18.04	3.20
19.73	18.04	16.49
19.73	18.04	11.41
19.73	18.04	2.13
19.73	18.04	1.17
19.73	18.04	17.31
19.73	18.04	0.60
19.73	18.04	2.54
19.73	18.04	4.89
19.73	18.04	1.31
19.73	18.04	1.91
19.73	18.04	2.79
19.73	18.04	1.56
19.73	18.04	16.51
19.73	18.04	-19.73
19.73	18.04	-0.11
19.73	18.04	6.50
19.73	18.04	2.87
19.73	18.04	6.04
19.73	18.04	7.84
19.73	18.04	14.43
19.73	18.04	8.45
19.73	18.04	10.72
19.73	18.04	10.21
19.73	18.04	3.56
19.73	18.04	17.54
19.73	18.04	2.36
19.73	18.04	10.84
19.73	18.04	18.62
19.73	18.04	1.12
19.73	18.04	2.90
19.73	18.04	11.16
19.73	18.04	1.35
19.73	18.04	2.77

19.73	18.04	2.29
19.73	18.04	3.76
19.73	18.04	5.47
19.73	18.04	-19.73
19.73	18.04	0.58
19.73	18.04	6.91
19.73	18.04	4.28
19.73	18.04	2.58
19.73	18.04	6.40
19.73	18.04	8.62
19.73	18.04	6.08
19.73	18.04	7.61
19.73	18.04	8.63
19.73	18.04	0.75
19.73	18.04	1.06
19.73	18.04	2.04
19.73	18.04	10.33
19.73	18.04	7.93
19.73	18.04	17.23
19.73	18.04	5.59
19.73	18.04	17.62
19.73	18.04	20.67
19.73	18.04	1.29
19.73	18.04	17.38
19.73	18.04	-3.71
19.73	18.04	3.42
19.73	18.04	10.03
19.73	18.04	6.55
19.73	18.04	0.00
19.73	18.04	0.28
19.73	18.04	0.88
19.73	18.04	0.59
19.73	18.04	13.86
19.73	18.04	0.42
19.73	18.04	0.31
19.73	18.04	-0.06
19.73	18.04	2.68
19.73	18.04	2.12
19.73	18.04	3.95
19.73	18.04	2.12
19.73	18.04	0.35
19.73	18.04	0.24

19.73	18.04	0.29
19.73	18.04	-0.09
19.73	18.04	2.83
19.73	18.04	1.91
19.73	18.04	2.63
19.73	18.04	8.55
19.73	18.04	5.03
19.73	18.04	4.69
19.73	18.04	5.15
19.73	18.04	7.65
19.73	18.04	1.29
19.73	18.04	6.24
19.73	18.04	2.58
19.73	18.04	5.76

48hr

dCt 48hr Infected (Infected Cq - Reference Cq)	ddCt (Infected dCt - Uninfected dCt)
0.25	-0.09
2.33	0.70
1.43	-0.39
3.18	-0.01
20.02	3.53
12.40	0.99
2.01	-0.12
1.39	0.22
17.12	-0.19
0.74	0.14
2.22	-0.32
4.25	-0.64
1.24	-0.07
2.10	0.19
3.05	0.27
1.78	0.22
13.96	-2.55
-18.04	1.70
0.13	0.24
6.21	-0.29
2.66	-0.22
6.39	0.35
7.63	-0.20
15.07	0.64
8.78	0.33
11.98	1.26
11.32	1.11
2.77	-0.79
19.51	1.97
2.22	-0.14
10.42	-0.42
19.35	0.73
1.17	0.05
2.49	-0.41
11.35	0.19
0.67	-0.68
2.35	-0.43

3.38	1.10
4.10	0.34
5.66	0.19
-18.04	1.70
0.26	-0.32
6.46	-0.45
4.19	-0.09
2.61	0.03
6.49	0.09
9.08	0.46
6.32	0.24
7.79	0.19
9.17	0.53
0.64	-0.11
1.11	0.05
3.45	1.41
10.48	0.15
8.22	0.29
15.26	-1.97
6.17	0.58
17.50	-0.12
15.69	-4.98
1.50	0.21
16.31	-1.07
-3.54	0.16
3.83	0.41
8.61	-1.41
6.40	-0.15
0.00	0.00
0.91	0.63
0.97	0.09
0.32	-0.27
13.54	-0.32
0.16	-0.26
0.30	-0.02
0.07	0.13
3.05	0.37
3.46	1.34
4.08	0.12
3.69	1.57
0.28	-0.07
0.57	0.34

0.93	0.65
-0.06	0.02
3.06	0.23
2.17	0.26
2.44	-0.19
8.94	0.39
5.54	0.51
5.32	0.63
5.36	0.21
7.69	0.04
1.14	-0.14
6.16	-0.09
2.89	0.31
5.85	0.09

**Expression Fold Change (2^ -ddCt)**		
1.07		
0.62	**Reference Gene**	SNORD49A (Diff = 1.91)
1.31		
1.01		
0.09		
0.50		
1.09		
0.86		
1.14		
0.91		
1.25		
1.56		
1.05		
0.88		
0.83		
0.86		
5.86		
0.31		
0.85		
1.23		
1.16		
0.79		
1.15		
0.64		
0.80		
0.42		
0.46		
1.73		
0.25		
1.10		
1.34		
0.60		
0.97		
1.32		
0.87		
1.60		
1.34		

0.47		
0.79		
0.87		
0.31		
1.25		
1.36		
1.06		
0.98		
0.94		
0.73		
0.84		
0.88		
0.69		
1.08		
0.96		
0.38		
0.90		
0.82		
3.92		
0.67		
1.09		
31.62		
0.87		
2.11		
0.89		
0.75		
2.66		
1.11		
1.00		
0.65		
0.94		
1.20		
1.25		
1.20		
1.01		
0.91		
0.77		
0.40		
0.92		
0.34		
1.05		
0.79		

0.64		
0.98		
0.85		
0.84		
1.14		
0.76		
0.70		
0.65		
0.87		
0.97		
1.10		
1.06		
0.81		
0.94		

miRNA Name	Fold Change (MDA-MB-231)
hsa-miR-143-3p	-2.40
hsa-miR-202-3p	3.92
hsa-miR-145-5p	-2.16
hsa-miR-181a-5p	-2.14
hsa-miR-206	31.62
hsa-miR-20b-5p	2.11
hsa-miR-214-3p	2.66
hsa-miR-200a-3p	-1.99
hsa-let-7f-5p	0.80
hsa-miR-146a-5p	1.73
hsa-miR-222-3p	0.83
hsa-miR-223-3p	0.80

NOTE: All expression fold changes recorded on this page are the manually calculated values

Qiagen Human Cancer Focus miRNA PCR Panel Expressi[illegible]	
MDA-MB-231	
**Qiagen Analysis Tool Results**	
**miRNA Name**	**Fold Change**
hsa-let-7a-5p	1.07
hsa-let-7b-5p	-1.63
hsa-let-7c-5p	1.31
hsa-let-7d-5p	1.01
hsa-miR-200a-3p	-1.99
hsa-let-7g-5p	1.09
hsa-let-7i-5p	-1.17
hsa-miR-1-3p	1.14
hsa-miR-100-5p	-1.10
hsa-let-7f-5p	1.25
hsa-miR-101-3p	1.56
hsa-miR-103a-3p	1.05
hsa-miR-106a-5p	-1.14
hsa-miR-106b-5p	-1.20
hsa-miR-107	-1.16
hsa-miR-125b-5p	-1.18
hsa-miR-126-3p	1.23
hsa-miR-130a-3p	1.16
hsa-miR-132-3p	-1.27
hsa-miR-10b-5p	1.15
hsa-miR-133a-3p	-1.56
hsa-miR-141-3p	-1.26
hsa-miR-143-3p	-2.40
hsa-miR-145-5p	-2.16
hsa-miR-146a-5p	1.73
hsa-miR-26a-5p	1.10
hsa-miR-150-5p	1.34
hsa-miR-155-5p	-1.66
hsa-miR-15a-5p	-1.04
hsa-miR-15b-5p	1.32
hsa-miR-149-3p	-1.14
hsa-miR-16-5p	1.60
hsa-miR-17-5p	1.34
hsa-miR-181a-5p	-2.14
hsa-miR-181b-5p	-1.27
hsa-miR-182-5p	-1.14
hsa-miR-27a-3p	1.25
hsa-miR-186-5p	1.36
hsa-miR-18a-5p	1.06

hsa-miR-191-5p	-1.02
hsa-miR-192-5p	-1.06
hsa-miR-148a-3p	-1.38
hsa-miR-194-5p	-1.18
hsa-miR-195-5p	-1.14
hsa-miR-196a-5p	-1.45
hsa-miR-19a-3p	1.08
hsa-miR-19b-3p	-1.04
hsa-miR-200b-3p	-1.11
hsa-miR-200c-3p	-1.22
hsa-miR-202-3p	3.92
hsa-miR-10a-5p	-1.49
hsa-miR-205-5p	1.09
hsa-miR-206	26.06
hsa-miR-20a-5p	-1.15
hsa-miR-20b-5p	2.11
hsa-miR-21-5p	-1.12
hsa-miR-210-3p	-1.33
hsa-miR-214-3p	2.66
hsa-miR-215-5p	1.11
SNORD38B (hsa)	1.00
hsa-miR-22-3p	-1.55
hsa-miR-221-3p	-1.06
hsa-miR-222-3p	1.20
hsa-miR-223-3p	1.25
U6 snRNA	1.37
hsa-miR-23a-3p	1.20
hsa-miR-23b-3p	1.01
hsa-miR-24-3p	-1.10
hsa-miR-25-3p	-1.30
hsa-miR-26b-5p	-1.09
hsa-miR-27b-3p	1.05
hsa-miR-29a-3p	-1.26
hsa-miR-29b-3p	-1.56
hsa-miR-29c-3p	-1.02
hsa-miR-30b-5p	-1.17
hsa-miR-30c-5p	-1.20
hsa-miR-30d-5p	1.14
hsa-miR-31-5p	-1.31
hsa-miR-34a-5p	-1.42
SNORD49A (hsa)	1.16
hsa-miR-423-5p	-1.55
hsa-miR-7-5p	-1.16
hsa-miR-9-5p	-1.03
hsa-let-7e-5p	1.10
hsa-miR-92b-3p	1.06

hsa-miR-93-5p	-1.24
hsa-miR-99a-5p	-1.06

ɔn-Fold Change Results (48hr B31-Infected/Uninfected Sam

ple)

Upregulated miRNAs		
Position	Symbol	Fold change
A01	hsa-let-7a-5p	1.07
A03	hsa-let-7c-5p	1.31
A04	hsa-let-7d-5p	1.01
A07	hsa-let-7g-5p	1.09
A09	hsa-miR-1-3p	1.14
A11	hsa-let-7f-5p	1.25
A12	hsa-miR-101-3p	1.56
B01	hsa-miR-103a-3p	1.05
B08	hsa-miR-126-3p	1.23
B09	hsa-miR-130a-3p	1.16
B11	hsa-miR-10b-5p	1.15
C04	hsa-miR-146a-5p	1.73
C06	hsa-miR-26a-5p	1.1
C07	hsa-miR-150-5p	1.34
C10	hsa-miR-15b-5p	1.32
C12	hsa-miR-16-5p	1.6
D01	hsa-miR-17-5p	1.34
D06	hsa-miR-27a-3p	1.25
D07	hsa-miR-186-5p	1.36
D08	hsa-miR-18a-5p	1.06
E03	hsa-miR-19a-3p	1.08
E08	hsa-miR-202-3p	3.92
E10	hsa-miR-205-5p	1.09
E11	hsa-miR-206	26.06
F01	hsa-miR-20b-5p	2.11
F04	hsa-miR-214-3p	2.66
F05	hsa-miR-215-5p	1.11
F06	SNORD38B (hsa)	1
F09	hsa-miR-222-3p	1.2
F10	hsa-miR-223-3p	1.25
F11	U6 snRNA	1.37
F12	hsa-miR-23a-3p	1.2
G01	hsa-miR-23b-3p	1.01
G07	hsa-miR-27b-3p	1.05
H01	hsa-miR-30d-5p	1.14
H04	SNORD49A (hsa)	1.16
H08	hsa-let-7e-5p	1.1
H09	hsa-miR-92b-3p	1.06

Downregulated m	
Position	Symbol
A02	hsa-let-7b-5p
A06	hsa-miR-200a-3p
A08	hsa-let-7i-5p
A10	hsa-miR-100-5p
B02	hsa-miR-106a-5p
B03	hsa-miR-106b-5p
B04	hsa-miR-107
B07	hsa-miR-125b-5p
B10	hsa-miR-132-3p
B12	hsa-miR-133a-3p
C01	hsa-miR-141-3p
C02	hsa-miR-143-3p
C03	hsa-miR-145-5p
C08	hsa-miR-155-5p
C09	hsa-miR-15a-5p
C11	hsa-miR-149-3p
D02	hsa-miR-181a-5p
D03	hsa-miR-181b-5p
D04	hsa-miR-182-5p
D09	hsa-miR-191-5p
D10	hsa-miR-192-5p
D11	hsa-miR-148a-3p
D12	hsa-miR-194-5p
E01	hsa-miR-195-5p
E02	hsa-miR-196a-5p
E04	hsa-miR-19b-3p
E06	hsa-miR-200b-3p
E07	hsa-miR-200c-3p
E09	hsa-miR-10a-5p
E12	hsa-miR-20a-5p
F02	hsa-miR-21-5p
F03	hsa-miR-210-3p
F07	hsa-miR-22-3p
F08	hsa-miR-221-3p
G02	hsa-miR-24-3p
G03	hsa-miR-25-3p
G05	hsa-miR-26b-5p
G08	hsa-miR-29a-3p
G09	hsa-miR-29b-3p
G10	hsa-miR-29c-3p
G11	hsa-miR-30b-5p
G12	hsa-miR-30c-5p
H02	hsa-miR-31-5p
H03	hsa-miR-34a-5p

H05	hsa-miR-423-5p
H06	hsa-miR-7-5p
H07	hsa-miR-9-5p
H10	hsa-miR-93-5p
H11	hsa-miR-99a-5p

miRNAs
Fold Change
-1.63
-1.99
-1.17
-1.1
-1.14
-1.2
-1.16
-1.18
-1.27
-1.56
-1.26
-2.4
-2.16
-1.66
-1.04
-1.14
-2.14
-1.27
-1.14
-1.02
-1.06
-1.38
-1.18
-1.14
-1.45
-1.04
-1.11
-1.22
-1.49
-1.15
-1.12
-1.33
-1.55
-1.06
-1.1
-1.3
-1.09
-1.26
-1.56
-1.02
-1.17
-1.2
-1.31
-1.42

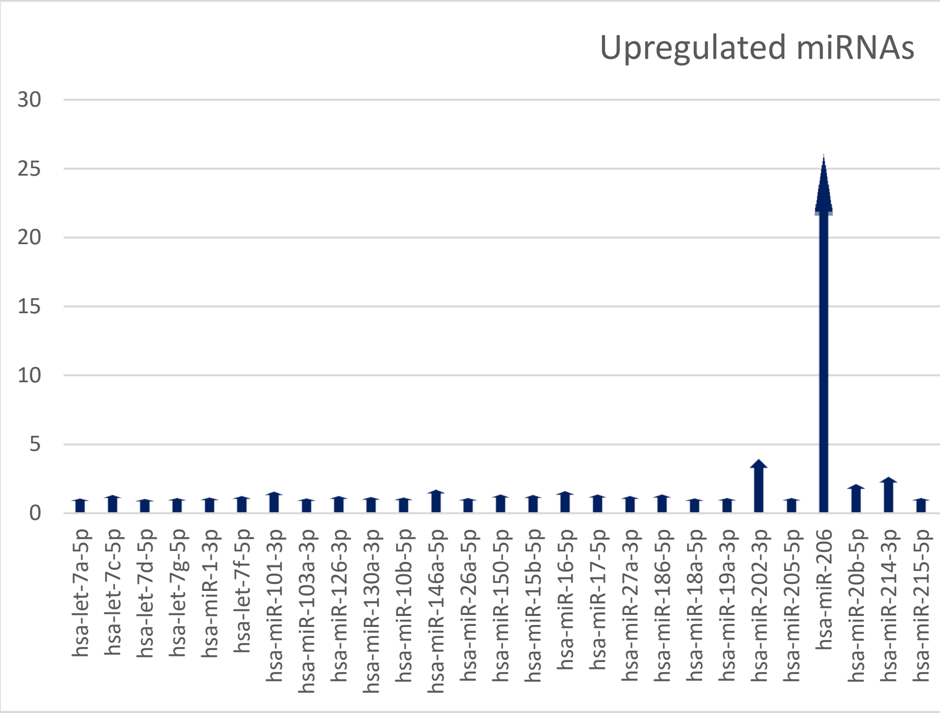

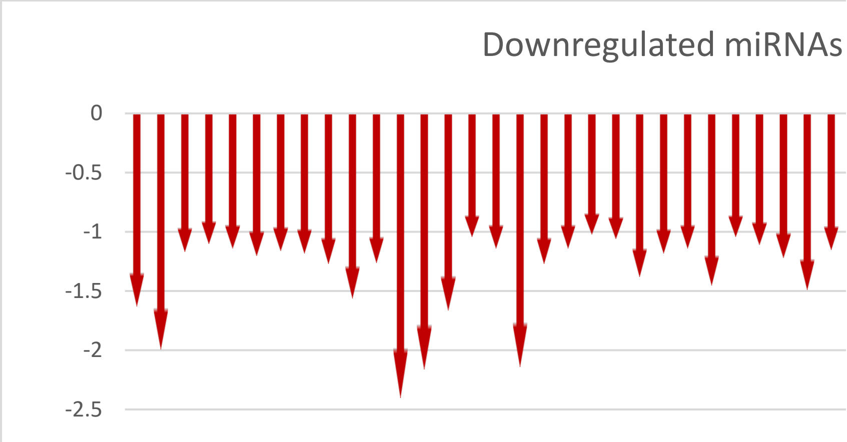

-1.55
-1.16
-1.03
-1.24
-1.06

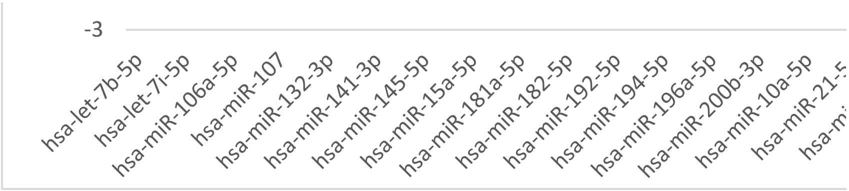

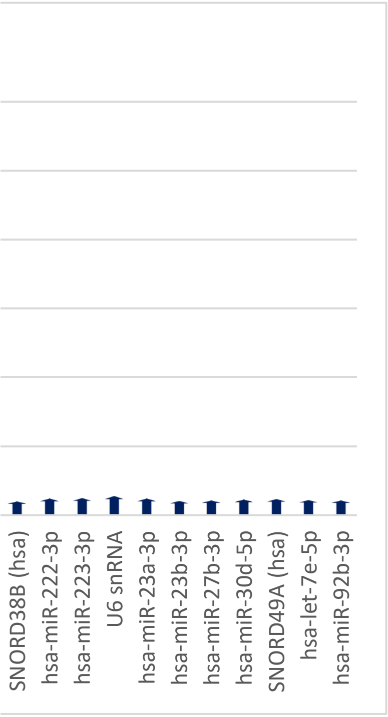
SNORD38B (hsa)
hsa-miR-222-3p
hsa-miR-223-3p
U6 snRNA
hsa-miR-23a-3p
hsa-miR-23b-3p
hsa-miR-27b-3p
hsa-miR-30d-5p
SNORD49A (hsa)
hsa-let-7e-5p
hsa-miR-92b-3p

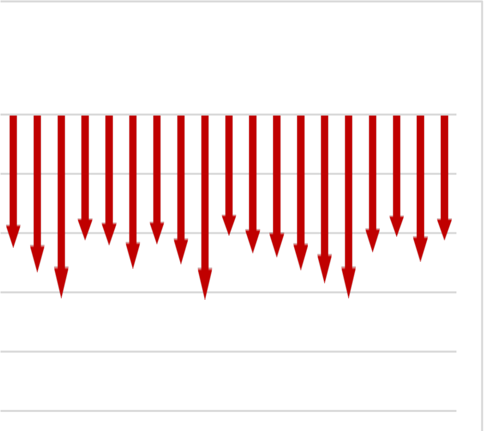

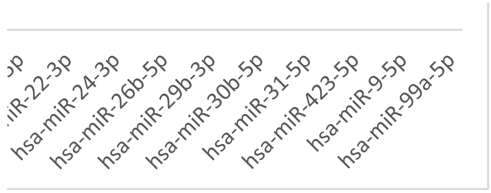
hsa-miR-24-3p
hsa-miR-26b-5p
hsa-miR-29b-3p
hsa-miR-30b-5p
hsa-miR-31-5p
hsa-miR-423-5p
hsa-miR-9-5p
hsa-miR-99a-5p
